# Dissecting exosome inhibitors: therapeutic insights into small-molecule chemicals against cancer

**DOI:** 10.1038/s12276-022-00898-7

**Published:** 2022-11-29

**Authors:** Jong Hyun Kim, Chan-Hyeong Lee, Moon-Chang Baek

**Affiliations:** 1 Department of Biochemistry, School of Medicine, Daegu Catholic University, Daegu, 42472 South Korea; 2 Department of Molecular Medicine, CMRI, Exosome Convergence Research Center (ECRC), School of Medicine, Kyungpook National University, Daegu, 41944 South Korea

**Keywords:** Cell biology, Cancer, Biologics

## Abstract

Intensive research in the field of cancer biology has revealed unique methods of communication between cells through extracellular vesicles called exosomes. Exosomes are released from a broad spectrum of cell types and serve as functional mediators under physiological or pathological conditions. Hence, blocking the release of exosome bio carriers may prove useful for slowing the progression of certain types of cancers. Therefore, efforts are being made to develop exosome inhibitors to be used both as research tools and as therapies in clinical trials. Thus, studies on exosomes may lead to a breakthrough in cancer research, for which new clinical targets for different types of cancers are urgently needed. In this review, we briefly outline exosome inhibitors and discuss their modes of action and potential for use as therapeutic tools for cancer.

## Cancer: Blocking vesicle release from tumors

Understanding how cells produce and release exosomes, nano-sized membrane-bound vesicles containing biological molecules, holds promise for developing new cancer therapies. Tumors release exosomes that remodel the nearby environment to promote cancer growth, suppress the immune response and induce drug resistance. Although some aspects of exosomes have been studied, little attention has been paid to exosome inhibitors and how they work. Moon-Chang Baek at Kyungpook National University in Daegu, South Korea, and co-workers have reviewed exosome inhibitors and possible applications to cancer, reporting that several compounds, such as pantethine, suppress exosome production and others, such as sulfisoxazole and macitentan, suppress exosome release. They report that inhibiting exosome release can prevent tumor cells from purging anti-cancer drugs, thus increasing drug accumulation and efficacy. This review highlights new options for alternative therapies for many types of cancer.

## Introduction

Exosomes are small nanosized extracellular vesicles (EVs) generated by the fusion of multivesicular bodies (MVBs) with the plasma membrane in eukaryotic cells and released under physiological and pathological conditions^1–6^. Exosomes secreted from cells were initially thought to be cellular waste resulting from cell damage or byproducts of cell homeostatic processes that exerted no significant impact on cell communication^7–10^. However, recent reports have shown that these proteins transport important signaling molecules involved in cell communication, migration, and numerous physiological processes^11–13^.

In cancer progression, tumor-derived exosomes have been closely associated with promoted tumor growth, increased abnormal angiogenesis, attenuated apoptosis, and even immune escape in proximal or distant microenvironments. During cancer metastasis, exosomes have been implicated in increasing metastatic cell migration and vascular invasion from the premetastatic niche^14,15^. Recently, chemoresistance, a state in which cancer cells resist chemotherapy drugs, has become a major challenge in cancer treatment and is known to be acquired through functional exosomes secreted from neighboring cancer cells^16–18^.

Moreover, a recent study reported that exosomes containing immune checkpoint proteins, such as PD-L1, on their surface are secreted, contributing to immunosuppression^19,20^. Exosomal PD-L1 binds to PD-1 on T cells, similar to cellular PD-L1, to inhibit the T-cell activity or attenuate the efficacy of PD-1/PD-L1 blockade therapy by binding to anti-PD-L1^21,22^. In addition, exosomes can contribute to immune evasion by transferring PD-L1 to distant cancer cells. PD-L1-enriched exosomes derived from breast cancer cells with high PD-L1 levels have been shown to increase PD-L1 levels by transferring PD-L1 to breast cancer cells with low PD-L1 expression^23^. Furthermore, the transfer of PD-L1 to macrophages by exosomal PD-L1 induces macrophage-mediated inhibition of CD8+ T-cell activity^24^. In addition to PD-L1, exosomes have been reported to transfer tumor-derived antigens that contribute to lymph node remodeling and immunosuppression through exosome uptake by lymphatic endothelial cells^25^. Therefore, the effect of exosomes on antitumor immunity is important, and the regulation of exosomes in immunotherapy is a new therapeutic target.

**Fig. 1:**
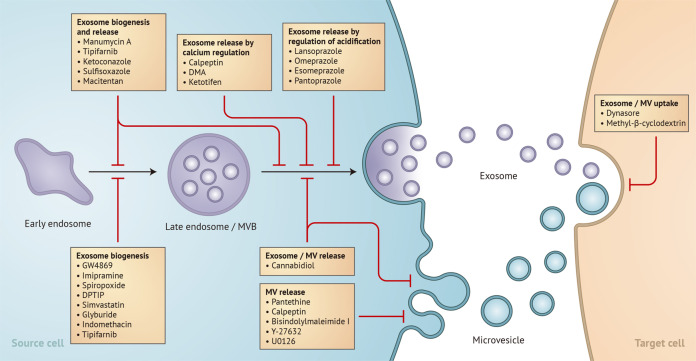
Exosome inhibitors affect different stages in exosome biogenesis. Exosomes are generated from endosomes, which are formed by the inward budding of early multivesicular body (MVB) membranes. These cells undergo a series of changes into late MVBs, which can be directed for lysosomal degradation or fusion with the plasma membrane, which leads to the release of exosomes or microvesicles. This image shows potential inhibitors that attenuate exosome biogenesis or release in the exosome manufacturing process.

To date, many studies have been published on bona fide exosomes under diverse physiological conditions, and many exosome inhibitors that disrupt different stages of biogenesis and release from cells have been discovered^3,6^. However, only a few reviews have been published to date, highlighting the significant roles of exosome inhibitors and their classification. In this review, the characteristics and physical properties of major drug inhibitors targeting specific mechanisms in the biogenesis and release of exosomes are discussed (Fig. 1). Furthermore, the use of exosome inhibitors in the treatment of various types of cancers exhibits great potential for developing effective therapeutic strategies in the future.

## Biogenesis of exosomes

Biogenesis of exosomes occurs via the fusion of late endosomes or MVBs with the plasma membrane. Early endosomes mature into late endosomes (MVBs), and in the process, intraluminal vesicles (ILVs) are formed by the invagination of the endosomal membrane. These MVBs may either subsequently fuse with lysosomes, leading to ILV destruction, or fuse with the cell membrane, releasing ILVs into the extracellular space. These secreted ILVs are known as exosomes^26^. MVB biogenesis is realized by the endosomal sorting complexes required for transport (ESCRT) machinery via two distinct mechanisms, the ESCRT-dependent or ESCRT-independent pathways^27–31^.

The ESCRT-dependent pathway requires ESCRT protein complexes with associated proteins to facilitate the generation of ILVs^32–34^. The ESCRT protein complex comprises multimolecular machinery consisting of four main complexes, ESCRT-0, -I, -II, and -III, and some additional associated proteins, namely, ALIX, VPS4, and ATPase. ESCRT-0 and -I interact with ubiquitylated transmembrane cargos, form microdomains in the endosomal membrane, and recruit ESCRT-III. ESCRT-III contains either ESCRT-II or ALIX. The polymerization of these components causes membrane neck constriction and cleavage to form ILVs. VPS4 and ATPase disassemble ESCRT, allowing it to be recycled. Thus, the ESCRT pathway can be recruited via other exosome biogenesis mechanisms.

On the other hand, the ESCRT-independent pathway involves lipid rafts inside endosomal membranes^35–39^. These lipid rafts contain high levels of cholesterol and sphingolipids, primarily sphingomyelin, which are produced by the neutral sphingomyelinase (nSMase) family. The enzyme nSMase converts sphingomyelin to ceramide, a cone-shaped rigid lipid. Ceramide is associated with the formation of microdomains that drive ILV formation from MVBs. The transport of MVBs to the cell membrane is dependent on interactions with actin and the microtubules of the cytoskeleton. Here, we discuss the key drugs that have been evaluated to date for blocking exosome biogenesis (Table 1).

**Table 1:** Exosome biogenesis inhibitors.

Name	Chemical Structure	Molecular Weight	Molecular Target	Biological Effects
Pantethine		554.72	Coenzyme A	- Regulation of lipid or cholesterol metabolism - Reduction in microvesicle (MV) release from breast cancer cells
GW4869		577.50	Neutral sphingomyelinase (nSMase)	- Neutral sphingomyelinase (nSMase) inhibitors - Inhibition of exosomes generation and MV shedding
Imipramine		280.19	Acid sphingomyelinase (aSMase)	- Acid sphingomyelinase (aSMase) inhibitors - Regulation of membrane fluidity, exosomes release, and MV generation
Spiroepoxide		378.46	Neutral sphingomyelinase (nSMase)	- Natural product, irreversible inhibitors of neutral sphingomyelinase (nSMase)
DPTIP		378.44	Neutral sphingomyelinase (nSMase 2)	- Neutral sphingomyelinase 2 (nSMase) inhibitor with nanomolar potency
Simvastatin		418.56	HMG-CoA reductase	- HMG-CoA reductase inhibitor - Regulation of lipid, including cholesterol, metabolism
Glibenclamide (Glyburide)		494.00	ATP-binding cassette transporter	- Inhibition of ATP-binding cassette transporter - Regulation of potassium channels
Indomethacin		357.78	Cyclooxygenase I and II	- Nonsteroidal anti-inflammatory drug - Regulation of ABCA3 or transporter and lipid transporter

The biological characteristics and structures of exosome inhibitors that inhibit exosome biogenesis.

## Exosome biogenesis inhibitors

### Pantethine

Pantethine is a pantothenic acid (vitamin B5) derivative used as an intermediate in the production of coenzyme A and plays a role in cholesterol metabolism^40^. Cholesterol is important for maintaining the fluidity of the cell membrane during membrane lipid bilayer reorganization. Therefore, pantethine can be used to disrupt the shedding of microvesicles (MVs). Previously, it had been shown to block the translocation of phosphatidylserine to the outer membrane leaflet, a fundamental process for MV production. A study by Roseblade et al. on breast cancer showed that pantethine reduced overall MV shedding by 24% in MCF-7 breast cancer cells^41^. Kavian et al. examined the role of MVs in systemic sclerosis (SSc), where endothelial cells (ECs) and fibroblasts are damaged and studied the effect of pantethine in preclinical in vivo SSc mouse models. The number of MVs released from ECs was decreased in a concentration-dependent manner, indicating that the addition of high concentrations of pantethine (50 μM and 100 μM) inhibited MV release. Moreover, pantethine substantially reduced the quantity of circulating EC-released MVs in these mice. In addition, oral administration of 150 mg/kg pantethine attenuated fibrosis and vascular alterations^42^.

### GW4869

GW4869 is a cell-permeable, selective compound that acts as a potent noncompetitive inhibitor of the membrane enzyme nSMase^43^. nSMase is a ubiquitous enzyme that generates bioactive lipid ceramides through hydrolysis of the membrane lipid sphingomyelin. GW4869 is the most widely used pharmacological agent to block exosome generation and reduce exosome release. The cone-shaped structure of ceramide released via nSMase activity is important for creating the large lipid raft domains involved in exosome shedding during the formation of spontaneous membrane curvature^36^. As a result, inhibition of nSMase reduces the number of exosomes released. Therefore, nSMase is a potential therapeutic target for the inhibition of exosome release. Many efforts have been made to develop nSMase inhibitors as exosome-inhibiting agents because exosomes have been implicated in tumor progression. Matsumoto et al. reported that GW4869 significantly decreased the growth of B16BL6 melanoma cells compared to untreated cells, indicating that inhibition of exosome secretion by GW4869 suppressed the autocrine-regulated proliferation of murine melanoma cells. The same effect was observed in mice injected subcutaneously with B16BL6 cells. In this study, B16BL6-derived exosomes promoted tumor growth in mice treated with PBS, whereas GW4869 treatment significantly reduced tumor growth and increased murine survival^36^. Panigrahi et al. reported that exosomes were secreted at higher levels in prostate cancer (PC) cells (LNCaP, 22Rv1, and PC3) under hypoxic conditions than under normoxic conditions, indicating their roles in a survival mechanism to remove metabolic waste, including lactic acid encapsulated in exosomes. Taken together, these studies showed that the inhibition of exosome secretion with GW4869 reduced cell viability, supporting the supposition suggestion of the fundamental role that exosomes play in the survival of these cancer cells^44^.

### Imipramine

Imipramine, a tricyclic antidepressant also known as a cyclic antidepressant, was introduced in the late 1950s. It inhibits the activity of acid sphingomyelinase (aSMase), which catalyzes the hydrolysis of sphingomyelin to ceramide, a process involved in membrane fluidity, exosome release, and MV generation^45^. Upon uptake into cells (especially inside endosomes and lysosomes), basic imipramine becomes protonated. In this form, it stimulates the proteolytic degradation of aSMase, which is then detached from the membrane owing to the loss of its negative charge. Thus, imipramine prevents the translocation of aSMase, thereby inhibiting MV and exosome secretion^46^. Kosgodage et al. demonstrated that 25 μM imipramine induced a 77% reduction in total EV release (both exosomes and MVs) from PC3 cells, although the separate effect of imipramine on exosomes and MVs was not tested. Imipramine can also be used to inhibit micropinocytosis^47^. However, further in-depth studies are required to investigate the role of imipramine at subtoxic concentrations and to characterize these vesicles extensively.

### Spiroepoxide

Spiroepoxide, a natural product, is a potent and selective irreversible inhibitor of nSMase^48^. This compound is known to block exosome release. Similar to GW4869, spiroepoxide at a 5 μM concentration has been shown to inhibit exosome release by 20%; however, the structure of this compound is entirely different from that of GW4869. Spiroepoxide has only one benzene ring and one ternary ring, whereas GW4869 has three benzene rings and two diazole groups.

### DPTIP (2,6-dimethoxy-4–(5-phenyl-4-thiophen-2-yl-1H-imidazole-2-yl)-phenol)

DPTIP is the most potent neutral sphingomyelinase 2 (nSMase 2) inhibitor, and the first inhibitor described with nanomolar potency (Rojas et al. 2018). The IC_50_ of DPTIP has been reported to be 30 nM, which indicates that it is more potent than the prototype inhibitor GW4869 (1 μM). Figuera-Losada et al. utilized DPTIP as a brain penetrant inhibitor to treat brain injury, and its hydroxyl group was shown to inhibit exosome release. Moreover, DPTIP has been shown to inhibit exosome release in a dose-dependent manner, decreasing exosome release by 50% in astrocytes at a high concentration of 30 μM^49^. Therefore, the inhibitory effect of DPTIP on exosome release strengthens the argument for its use as a potential agent for the synergistic treatment of cancer in the future.

### Simvastatin

Simvastatin, an HMG-CoA reductase inhibitor, prevents cholesterol synthesis and is often used to decrease elevated lipid levels. Since cholesterol is an integral constituent of endosomal membranes, simvastatin has been shown to significantly reduce exosome secretion in both epithelial cells and monocytes^50^. However, the effect of simvastatin in cancer cell lines has not been tested. However, simvastatin has been shown to inhibit endocytosis in a dose-dependent manner and decrease intracellular proteins associated with exosomes, such as ALIX, CD63, and CD81.

### Glibenclamide (Glyburide)

Glibenclamide, known as glyburide, belongs to the sulfonylureas family and inhibits MV release through an ATP-binding cassette (ABC) transporter. It has also been used as an antidiabetic drug that inhibits the SUR receptor (a member of the ABC family), which is involved in insulin release via the regulation of potassium channels. Glibenclamide has been shown to interact with other proteins of the ABC family, which has been implicated by the recycling of cholesterol and phospholipids mediated by circulating apolipoprotein A1 (apoA-I) during their maturation. Since cholesterol plays a fundamental role in MV and exosome production, this compound can be used as a potential inhibitor of exosome release^51^. Henricksson et al. suggested that glibenclamide also has an anticoagulant effect on monocytes in vitro, indicating a reduction in MV release^52^. In contrast, Kosgodage et al. reported that glibenclamide did not affect MV release from prostate cancer (PC3) and breast cancer (MCF7) cells^47^.

### Indomethacin

Indomethacin, a member of the nonsteroidal anti-inflammatory drug (NSAID) family, is used to decrease prostaglandin production during inflammation. It has been shown to nonselectively inhibit cyclooxygenase I and II, as well as downregulate the transcription of the ABCA3 transporter, which is involved in lipid transport. Moreover, inhibition of ABCA3 has been shown to attenuate exosome release, as lipids are important for exosome biogenesis^53^. Koch et al. demonstrated that doxorubicin, a cytotoxic drug, was encapsulated in released exosomes. Treatment with 10 μM indomethacin inhibited the release of drug-containing exosomes, and this inhibition likely increased the cytotoxic effect on cancer cells due to their accumulation inside the cells^54^.

## Exosome release

Exosomes are known to be involved in diverse pathophysiological conditions related to disease development and progression^3,4,55^ and are released via various mechanisms involving protein kinases, calcium channels, Rab signaling, and ESCRT-dependent pathways. These pathways have been identified as targets for exosome release inhibition and may not be directly related to lipid-related pathways or cytoskeletal organization; hence, they are classified separately^56–59^. Many pharmacological agents that inhibit exosome release have been investigated both as research tools and as potential therapeutic agents. Here, we discuss the key drugs that have been evaluated to date for blocking exosome release (Table 2)^60–63^.

**Table 2:** Exosome release inhibitors.

Name	Chemical structure	Molecular Weight	Molecular Target	Biological Effects
Calpeptin		362.46	Calpain	- Cytosolic cysteine protease with the calcium-binding site - Inhibition of MV release from prostate cancer cells
Bisindolyl maleimide I		448.94	Protein kinase C (PKC)	- PKC inhibitors - Inhibition of MV release from prostate cancer cells
Y-27632		320.26	Rho-associated protein kinases (ROCK)	- ROCK inhibitors - Regulation of cytoskeletal re-organization
U0126		380.48	Mitogen-Activated Protein Kinase Kinase(MEK)	- Noncompetitive inhibitor of MEK1 and MEK2 - Inhibition of MV release from THP-1 or hMDM cells
Manumycin A		550.64	Ras and Farnesyl Transferase (FTase)	- Ras farnesyltransferases inhibitors - Involved in wound-healing processes
Dimethyl amiloride		294.14	Na^+^/Ca^++^ Channels	- Blocker of H+/Na+ and the Na+/Ca2+ channels regulating calcium level
Tipifarnib		489.40	Rab and Farnesyl Transferase (FTase)	- Rab farnesyltransferases inhibitors - decrease the level of Rab27A, Alix, and nSMase2 in cancer cells
Ketoconazole		531.43	Rab and Farnesyl Transferase (FTase)	- Less active than tipifarnib
Sulfisoxazole		267.30	Endothelin Receptor A	- First known as a competitive inhibitor of dihydropteroate synthetase - Targeting endothelin receptor A
Macitentan		588.28	Endothelin Receptor A/B	- Approved for the treatment of pulmonary arterial hypertension
Cannabidiol		314.47	Unknown	- Anxiolytic drug with phytocannabinoid
Ketotifen		309.42	Ca^++^ Channel	- Antihistamine blocking calcium channel

The biological characteristics and structures of exosome inhibitors decrease, decreasing exosome release or secretion.

## Exosome release inhibitors

### Calpeptin

Calpeptin is an inhibitor of a cysteine protease, calpain. Calpain is a heterodimer enzyme consisting of a large catalytic subunit (80 kDa) and a regulatory subunit (28 kDa) harboring a calcium-binding site. Calpeptin is a calcium-dependent neutral, cytosolic cysteine protease that is dysregulated in cancer cells and involved in various processes, including apoptosis, cell proliferation, migration, tumor invasion, and cancer progression. The calpain proenzyme is activated by calcium binding, which induces a conformational change, leading to self-cleavage and activation of the proenzyme. Active calpain has been found to regulate the activity of numerous protein substrates, including G-proteins and cytoskeletal proteins, thereby suggesting its role in promoting MV shedding through the regulation of cytoskeleton remodeling^62^. However, this regulatory activity of calpain is inhibited by calpeptin, which leads to a reduction in the release of MV from cells.

Calpeptin is a reversible semisynthetic peptidomimetic aldehyde inhibitor developed via the modification of a *Streptomyces* peptide aldehyde^64^. It has been extensively used as a microvesiculation inhibitor. Atanassoff et al. investigated the effects of calpeptin on MV release using streptolysin-O (known to damage cell membranes) in HEK293 cells and observed that calpeptin treatment led to streptolysin-O accumulation inside these cells and increased the rate of cell lysis^65^. Studying PC3 cells, Jorfi et al. reported that the inhibition of MV release by calpeptin resulted in the accumulation of the cytotoxic drugs docetaxel and methotrexate inside cells, which significantly decreased the cell proliferation and increased the cell death rates. Moreover, a subsequent preclinical experiment with an in vivo mouse model showed results similar to those obtained with the in vitro model, indicating that the combined treatment of docetaxel and calpeptin significantly attenuated tumor mass compared to the effect of treatment with docetaxel alone^66^. According to studies reported herein, the efficacy of exosome release inhibition by calpeptin is clear in experimental in vitro or in vivo mouse models. However, the selective effect of calpeptin on exosome release needs to be further evaluated on the basis of its concentration.

### Bisindolylmaleimide I

Bisindolylmaleimide I is a highly selective protein kinase C inhibitor that is known to be a cell-permeable and reversible competitive inhibitor. This inhibitor can attenuate the activity of different isoforms of protein kinase C (PKC), that is, α, βI, βII, γ, δ, and ε, in diverse cells. MV release is dependent on calcium and the externalization of lipids, both of which involve PKC activation by diacylglycerol. Stratton et al. demonstrated that MV release was inhibited by bisindolylmaleimide I, which blocked MV shedding in PC cells^63^.

### Y-27632

Rho-associated protein kinase (ROCK) is a serine-threonine kinase in the PKA/PKG/PKC family and is expressed in two isoforms: ROCK1 and ROCK2. ROCK controls the shape and movement of cells by regulating the cytoskeleton. Reorganization of the cytoskeleton by actin filaments is important for MV shedding. Y27632 is a cell-permeable, highly potent, competitive inhibitor of both ROCK1 and ROCK2, and it competes with ATP by interacting with the catalytic sites of these two kinases^67^. Li et al. investigated the role of ROCK in MV formation in diverse cancer cell lines, including triple-negative breast cancer (MDA-MB-231) and ovarian cancer (HeLa) cells, which express high levels of these kinases, and a substantial decrease in MV release was observed upon treatment of these cells with RhoA siRNA or the ROCK inhibitor Y-27632^58^. Sapet et al. also reported that MV release was inhibited by a ROCK inhibitor in a microvascular endothelial cell line (the HMEC-1 cell line) activated by thrombin, which was used to stimulate an increase in MV shedding by these cells^67^.

### U0126

U0126 is a highly selective noncompetitive inhibitor of MEK1 and MEK2, two protein kinases in the mitogen-activated protein kinase kinase (MAPKK) family. U0126 has been found to functionally antagonize AP-1 transcriptional activity via noncompetitive inhibition of the dual specificity kinase MEK, with an IC_50_ of 72 nM for MEK1 and 58 nM for MEK2. ERK, an upstream kinase of MEK, stimulates MV biogenesis, and its inhibition has been associated with MV shedding reduction. Mingzhen et al. demonstrated that MAPK activation by tobacco smoke extract increased the release of procoagulant MV from THP-1 monocytic cell lines and primary human monocyte-derived macrophages. Subsequently, it was observed that inhibition of MEK by U0126 reduced procoagulant MV release from these stimulated monocyte and macrophage cell lines^60^.

### Manumycin A

Ras is a small GTPase that contributes to the regulation of diverse proteins and many different cellular processes, including cytoskeletal rearrangement, cell adhesion, migration, proliferation, differentiation, apoptosis, and exosome release. In cancer cells, Ras activation is involved in the invasiveness, metastasis, and suppression of apoptosis. Ras consists of two domains: the G domain (binds to GTP) and the C-terminal domain (membrane-targeting domain). The C-terminal domain is important for membrane recruitment because it is modified by a lipid chain that affects membrane trafficking. Lipid modification involves farnesylation mediated by farnesyltransferase (FTase). When this modification is inhibited by a farnesyltransferase inhibitor (FTI), Ras activity is inhibited. Thus, the Ras-induced downstream signaling cascade is blocked in cells.

One of the most commonly used FTIs is manumycin A, a cell-permeable, selective, and potent inhibitor of Ras FTase. Thus, since one of the cellular functions of Ras is exosome release, manumycin A has been investigated to ascertain its inhibitory effect on exosome function, including exosome biogenesis release. Datta et al. investigated the effects of manumycin A on ESCRT-dependent exosome biogenesis in PC cells. Manumycin A administered at a 250 nM concentration, induced a reduction in exosome production by 50–60% in these cancer cells. Moreover, treatment with manumycin A and GW4869 (nSMase inhibitor) in combination further reduced exosome production in these cancer cells compared with either treatment alone, indicating that other proteins may be involved in additional pathways of exosome biogenesis^68^. Zhou et al. examined the role of exosomes in the wound-healing process of BUMPT cells. Exosome secretion in these cells was induced by scratching a cell monolayer in a wound-healing assay. The wound area increased upon treatment with manumycin A or GW4869, indicating that exosomes facilitate the wound healing process in BUMPT cells^69^. However, the limited number of studies on manumycin A reported to date is relevant to the conclusion that it inhibits exosome release. Future studies are necessary to determine the noncytotoxic concentration of this drug that is sufficient for inhibiting exosome release.

### Dimethyl amiloride

Dimethyl amiloride (DMA) is a derivative of amiloride used to treat high blood pressure. Intracellular calcium levels induce exosome release, and calcium homeostasis is maintained by calcium channels. Therefore, because DMA impairs the function of calcium channels, it has been proposed as a prospective exosome blocker^70^. Chalmin et al. reported that DMA inhibited the release of exosomes in various cell lines (CT26 colon carcinoma cells, EL4lymphoma cells, and H23lung adenocarcinoma cells) and different mouse models (C57BL/6, BALB/c, and nude mice)^71^.

### Tipifarnib

Tipifarnib is a potent FTI that interferes with cell growth and apoptosis. It was one of four compounds identified from 1280 pharmacologically active compounds by^72^. It inhibits the biogenesis and secretion of exosomes to slow cancer progression. It was observed that tipifarnib at concentrations ranging from 0.25 to 1 μM significantly decreased the number of released exosomes from aggressive prostate cancer (PC) CD63-GFP-expressing C4-2B cells, substantiating its inhibitory effect on exosome release. Moreover, tipifarnib decreased the levels of Rab27A, Alix, and nSMase2 in cancer cells but not in normal cells. This result is crucial for its clinical use as an exosome inhibitor^56,61,73^.

### Ketoconazole

Sella et al. reported ketoconazole to be an exosome release inhibitor after it was identified from compounds approved by the United States FDA for treating PC patients^74^. Ketoconazole (5 μM) decreased the levels of exosomes produced by C4-2B and PC3 cells and showed a robust dose-dependent decrease in RAB27A, Alix, and nSMase2 expression both in C4-2B and PC3 cells but not in RWPE-1 cells. The efficacy of ketoconazole has been observed to be lower than that of tipifarnib. Furthermore, both compounds carry the same components, namely diazole and aromatic moieties, suggesting the importance of both of these moieties in the inhibition of exosome release.

### Endothelin A receptor antagonist

Recently, endothelin A receptor (ETA) antagonists, such as sulfisoxazole (SFX) and macitentan (MAC), were discovered to be new agents for the inhibition of exosome secretion^75,76^. SFX is an orally administered FDA-approved drug that is not cytotoxic at effective doses. It was first identified as a competitive inhibitor of dihydropteroate synthetase, which generates dihydropteroic acid by condensing dihydropteroate diphosphate and p-aminobenzoic acid^77^. Im et al. showed that SFX efficiently reduced the release of small vesicles via an ESCRT-dependent pathway, as shown in in vitro and preclinical in vivo studies. SFX blocked exosome release and cancer cell activity by targeting ETA in breast cancer cells (MCF10A, MCF7, and MDA-MB-231 cells)^75^. Moreover, it decreased the expression of several Rab and ESCRT-related components, such as VPS4B and Alix, without affecting other vesicle-producing pathways, such as components in ESCRT-dependent pathways, or altering intracellular calcium levels.

Another ETA antagonist, MAC, has been reported to inhibit exosomal PD-L1 secretion^76^. MAC is an ETA/ETB dual antagonist approved by the FDA for the treatment of pulmonary arterial hypertension. Inhibition of exosomal PD-L1 secretion by MAC has been shown to reduce the number of exosomes that bind to PD-1 on T cells and to enhance antitumor immunity by inducing the reinvigoration of exhausted T cells. These results suggest that an effective drug can be developed by targeting different stages of exosome biogenesis and secretion.

### Cannabidiol

Cannabidiol (CBD), a phytocannabinoid derived from *Cannabis sativa* currently used as an anxiolytic drug with anti-inflammatory, antineoplastic, and analgesic properties, can inhibit exosome and MV release^78–81^. Kosgodage et al. reported that 5 μM CBD blocked exosome release by 50% and specifically decreased exosome release in diverse cancer cells (PC3 cells; liver cancer, HEPG2 cells; breast cancer, MDA-MB231 cells). These results indicate that this compound is a potential therapeutic agent for reducing EV^82,83^. The mode of action of CBD involves its interference with CD63 expression, as a lower CD63 expression level was observed after CBD treatment in three cancer cell lines.

### Ketotifen

Ketotifen, an antihistamine, is a store-operated calcium channel blocker that stabilizes mast cells^84^. Khan et al. reported that ketotifen (10 μM) decreased exosome release from various cancer cells (HeLa, MCF7, and BT549 cells) by 70%^85^. It has been shown to increase the sensitivity of cancer cells to doxorubicin and attenuate the progression of cancer cell survival. Moreover, it blocks calcium influx into cells, thereby inhibiting exosome release via calcium-dependent mechanisms, as calcium is required for exosome release. The mode of action of ketotifen is the blockade of calcium channels, which might be important for developing new potential strategies to inhibit exosome release^86,87^.

## Other inhibitors

Since the mechanism of exosome release is complex, it is not surprising that the same pathways can be impaired by different drugs targeting diverse proteins involved in the same signaling cascade. Therefore, although the inhibitors mentioned above are commonly used, other inhibitors have also been investigated for their ability to inhibit exosome release. Here, we describe other exosome inhibitors that have the potential to be used in future strategies (Table 3).

**Table 3:** Other inhibitors.

Name	Chemical Structure	Molecular Weight	Molecular Target	Biological Effects
Lansoprazole		369.36	V-H^+^-ATPase	- Proton pump inhibitor (PPI) - Inhibition of acidification - Inhibition of exosome release
Omeprazole		345.41	V-H^+^-ATPase	- Proton pump inhibitor (PPI) - Inhibition of acidification - Inhibition of exosome release
Esomeprazole		713.12	V-H^+^-ATPase	- Proton pump inhibitor (PPI) - Inhibition of acidification - Inhibition of exosome release
Pantoprazole		383.37	V-H^+^-ATPase	- Proton pump inhibitor (PPI) - Inhibition of acidification - Inhibition of exosome release
Dynasore		322.3	Dynamin	- Clathrin-dependent endocytosis inhibitors - Noncompetitive inhibitor of dynamin 1 and 2
Methyl-beta-cyclodextrin (MbCD)		1310	Caveolin	- Caveolin-mediated endocytosis inhibitors

The biological characteristics and structures of exosome inhibitors that are involved in decreasing exosome release via other mechanisms.

### Lansoprazole, omeprazole, esomeprazole, and pantoprazole

Exosome inhibition can be realized via multiple strategies that affect different mechanisms, note merely exosome biogenesis and exosome release discussed thus far in this review. Parolini et al. reported that exosome trafficking is regulated by microenvironmental pH in cancer cells^88^. Although exosome release is increased at a low pH, it is reduced under alkalizing conditions in tumor cells^89^. In cancer cells, proton transporter V-ATPase plays a key role in maintaining an alkaline intracellular pH and an acidic extracellular pH. Therefore, V-ATPase may be a potential target to block exosome release. A proton pump inhibitor (PPI) of V-ATPase is a therapeutic tool used for improving the anti-acidic properties of cancer cells because it can inhibit exosome release^90–94^. Federici et al. investigated the reduction in the number of exosomes released by a PPI in tumor cells and showed that the PPI lansoprazole caused a marked reduction in exosome release from human melanoma cells and showed the inhibition of plasmatic exosomes release by treatment with a PPI in human tumor cells in vivo^95^. Two previous studies also reported that pretreatment with PPIs such as omeprazole, esomeprazole, or pantoprazole induced the sensitivity of human solid tumors to cytotoxic drugs, indicating that these inhibitors (lansoprazole, omeprazole, esomeprazole, or pantoprazole) inhibited the acidification of the extracellular medium of cancer cells (melanoma, adenocarcinoma, and lymphoma cells) and can therefore be used to block exosome release^93,96^.

### Dynasore, and methyl-β-cyclodextrin (MβCD) (an inhibitor of proteins involved in endocytosis)

Exosomes are released via endocytosis, which involves two important proteins, clathrin and caveolin. Although dynasore is a widely used clathrin-dependent endocytosis (CDE) inhibitor, it exerts many nonspecific effects^97^. Dynasore, a noncompetitive inhibitor, inhibits the GTPase activity of dynamin 1 and dynamin 2^98^. Dynamin is necessary for the CDE process and regulates the assembly of actin filaments into bundles during endocytosis^99^. Dynasore inhibits the endocytosis of exosomes and exhibits some additional functions in exosome release from cells. However, it has not been extensively tested in cancer cells and neuronal cells since it had been reported that dynasore failed to inhibit the exocytosis of synaptic vesicles^100^.

Methyl-β-cyclodextrin (MβCD) inhibits caveolin-mediated endocytosis, which involves caveolin, a protein that regulates endocytosis. MβCD depletes cholesterol in the plasma membrane, disturbs lipid rafts, and subsequently diminishes the uptake of exosome-sized vesicles. This compound is not an exosome-specific inhibitor because it decreases micropinocytosis and exosome secretion from the cells. Kosgodage et al. reported that MβCD reduced exosome secretion but severely impaired cell morphology at high concentrations (5 mM), which was beyond the range used to inhibit exocytosis^47^. MβCD has also been used in combination with lovastatin (an inhibitor of cholesterol synthesis) to prevent cholesterol synthesis, leading to dramatically depleted levels of cholesterol in cells.

## Conclusion and further perspective

Although EVs, including exosomes, are constantly released by most mammalian cells, they had largely been considered to be byproducts of membrane biosynthesis and shedding^101,102^. However, their importance has increased since their critical biological function in cell-to-cell communication was discovered^103^. Intercellular communication via exosomes is evident throughout cancer progression and pathogenesis^104^. During tumorigenesis, exosomes can facilitate the programming of a tumoral microenvironment that favors early tumor initiation stages, and subsequently, they can regulate the immune response to enable tumor progression and induce tumor cell survival by promoting angiogenesis, metastasis, and drug resistance, thereby maintaining tumors^105–107^. The biogenesis and release of exosomes are regulated during cancer progression, and the exosomes released from normal cells and cancer cells are quantitatively and qualitatively different^108–113^. In addition to their roles in cancer pathogenesis, exosomes may help in developing two cancer management fields, specifically, the fields associated with noninvasive diagnostic tools for liquid biopsy and with therapeutic tools for immunotherapy, vaccine development, and drug delivery. Although preclinical studies have shown the therapeutic potential of exosomes in cancer treatment, many clinical trials have not led to successful outcomes, which has been attributed to major hurdles in understanding exosome biogenesis/release, protein sorting, isolation of pure exosomes, large-scale exosome production, and exosome transport.

Recently, a significant contribution of exosomal PD-L1 was reported; it lowered the response to immune checkpoint blockade (ICB) therapy, such as PD-1/PD-L1 blockade therapy^21–23^. Several studies demonstrated the potential of exosomal PD-L1 inhibition in enhancing immunotherapy responsiveness. Genetic inhibition of Rab27a, an exosome secretion regulator, or nSMase, which is involved in exosome biogenesis, significantly reduced tumor progression by suppressing exosomal PD-L1 activity^22,114^. It has also been reported that the genetic inhibition of ETA or LSD1, which are other exosome release-related genes, increased antitumor immunity^76,115^. A similar phenomenon has been reported after treatment with GW4869, which inhibits exosome secretion by inhibiting the activity of nSMase and showed a synergistic effect in combination with anti-PD-L1 therapy^114^. The FDA-approved ETA antagonists SFX and MAC have been reported to improve the efficacy of anti-PD-L1 treatment in breast, lung, and colon cancer models^76,116^. These results show the potential of exosome inhibitors as effective administrated agents for ICB therapy.

Although many in vitro, preclinical in vivo, and clinical studies have indicated that many compounds can inhibit the biogenesis and release of exosomes or EVs, more extensive research with both in vitro and in vivo models is required to investigate the activities of these compounds for the development of single or combination treatment strategies. These compounds target different proteins at different stages of exosome biogenesis and release. Furthermore, the importance of exosome inhibitors being used as synergistic agents for cancer therapy will eventually increase as research on exosome biology is further explored. Therefore, novel strategies targeting exosomes for attenuating pathological communication in cancer will show significant therapeutic potential in cancer patients in the future. Moreover, it is crucial to develop pharmacological compounds that can selectively reduce exosome biogenesis/release without inducing many side effects, and the consolidated overview provided in this review of the current research being carried out in this field may be a reference for future drug development.
